# Soluble expression of recombinant proteins in the cytoplasm of *Escherichia coli*

**DOI:** 10.1186/1475-2859-4-1

**Published:** 2005-01-04

**Authors:** Hans Peter Sørensen, Kim Kusk Mortensen

**Affiliations:** 1 Danish Technological Institute, Holbergsvej 10, 6000 Kolding, Denmark; 2 Laboratory of BioDesign, Department of Molecular Biology, Aarhus University, Gustav Wieds Vej 10C, 8000 Aarhus C, Denmark

## Abstract

Pure, soluble and functional proteins are of high demand in modern biotechnology. Natural protein sources rarely meet the requirements for quantity, ease of isolation or price and hence recombinant technology is often the method of choice. Recombinant cell factories are constantly employed for the production of protein preparations bound for downstream purification and processing. *Eschericia coli *is a frequently used host, since it facilitates protein expression by its relative simplicity, its inexpensive and fast high density cultivation, the well known genetics and the large number of compatible molecular tools available. In spite of all these qualities, expression of recombinant proteins with *E. coli *as the host often results in insoluble and/or nonfunctional proteins. Here we review new approaches to overcome these obstacles by strategies that focus on either controlled expression of target protein in an unmodified form or by applying modifications using expressivity and solubility tags.

## Introduction

Microorganisms like the enterobacterium *Escherichia coli *are outstanding factories for recombinant expression of proteins. An expression system for the production of recombinant proteins in *E. coli *usually involves a combination of a plasmid and a strain of *E. coli *[1]. The main purpose of recombinant protein expression is often to obtain a high degree of accumulation of soluble product in the bacterial cell. This strategy is not always accepted by the metabolic system of the host and in some situations a cellular stress response is encountered. Another response encountered in recombinant systems is the accumulation of target proteins into insoluble aggregates known as inclusion bodies. These aggregated proteins are in general misfolded and thus biologically inactive [2].

Under normal cellular conditions a subset of cytoplasmic proteins are able to fold spontaneously [3] while aggregation prone proteins require the existence of a number of molecular chaperones that interact reversibly with nascent polypeptide chains to prevent aggregation during the folding process [4]. Aggregation of recombinant proteins overexpressed in bacterial cells could therefore result either from accumulation of high concentrations of folding intermediates or from inefficient processing by molecular chaperones. No universal approach has been established for the efficient folding of aggregation prone recombinant proteins [1].

**Figure 1:**
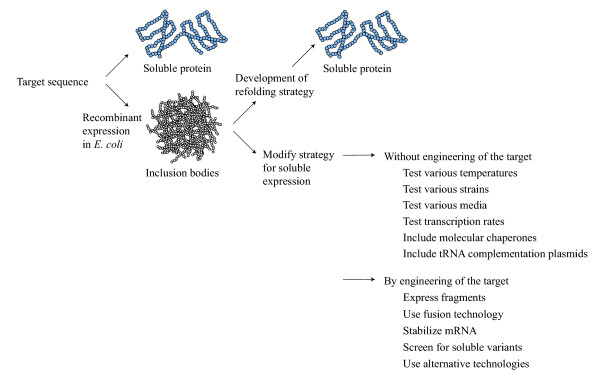
Downstream applications employed to obtain soluble proteins from recombinant *E. coli*. As a common trait the *in vivo *strategies aims at lowering the metabolic burden associated with recombinant expression. Some of the mentioned strategies have therefore merely indirect influence on folding such as the use of tRNA complementation plasmids and stabilization of mRNA (see text and ref [1] for details).

The literature describes a number of methods for the redirection of proteins from inclusion bodies into the soluble cytoplasmic fraction (Figure 1). Overall they can be divided into procedures where protein is refolded from inclusion bodies [5] and procedures where the expression strategy is modified to obtain soluble expression. In this review we focus on methods developed for soluble expression in the *E. coli *cytoplasm. Refolding from inclusion bodies is in many cases considered undesireable, but is however sometimes the method of choice. The major obstacles are the poor recovery yields, the requirement for optimization of refolding conditions for each target protein and the possibility that the resolubilization procedures could affect the integrity of refolded proteins. In addition, the purification of highly expressed soluble protein is less expensive and time consuming than refolding and purification from inclusion bodies. Maximizing the production of recombinant proteins in a soluble form is therefore an attractive alternative to *in vitro *refolding procedures. The methods used to mediate soluble expression can be divided into procedures where target modification is avoided and procedures where the target sequence is engineered (Figure 1).

## Strategies where target modification is avoided

Some proteins directly influence the cellular metabolism of the host by their catalytic properties, but in general expression of recombinant proteins induces a "metabolic burden". The metabolic burden is defined as the amount of resources (raw material and energy), which are withdrawn from the host metabolism for maintenance and expression of the foreign DNA [6]. The formation of inclusion bodies occurs as a response to the accumulation of denatured protein. The metabolic burden and inclusion body formation are not directly linked but are both among the main factors to determine the ability of cells to produce soluble recombinant protein. Since the accumulation of denatured protein and the metabolic burden can be controlled by a number of environmental factors, we are partially able to control the formation of soluble protein *in vivo*.

### Protein expression at reduced temperatures

A well known technique to limit the *in vivo *aggregation of recombinant proteins consists of cultivation at reduced temperatures [7]. This strategy has proven effective in improving the solubility of a number of difficult proteins including human interferon α-2, subtilisin E, ricin A chain, bacterial luciferase, Fab fragments, β-lactamase, rice lipoxygenase L-2, soybean lypoxygenase L-1, kanamycin nuclotidyltransferase and rabbit muscle glycogen phosphorylase (see [8] and references cited therein).

The aggregation reaction is in general favored at higher temperatures due to the strong temperature dependence of hydrophobic interactions that determine the aggregation reaction [9]. A direct consequence of temperature reduction is the partial elimination of heat shock proteases that are induced under overexpression conditions [10]. Furthermore, the activity and expression of a number of *E. coli *chaperones are increased at temperatures around 30°C [11,12]. The increased stability and potential for correct folding at low temperatures are partially explained by these factors.

However, a sudden decrease in cultivation temperature inhibits replication, transcription and translation [13]. Traditional promoters used in vectors for recombinant protein expression are also strongly affected in terms of efficiency [14]. A similar transcriptional effect is achieved when a moderately strong or weak promoter is used or when a strong promoter is partially induced. Low induction levels have been found to result in higher amounts of soluble protein [15]. This is a result of the reduction in cellular protein concentration which favors folding. However, bacterial growth is decreased, thus resulting in a decreased amount of biomass.

Different strategies aimed at optimizing the expression of recombinant proteins at low temperature are as follows.

A system based on the *cspA *promoter was developed for the expression of proteins at low temperature [16]. The *cspA *promoter is highly induced at low temperature and is well repressed at and above 37°C. A sequence encoding the TolAI-β-lactamase fusion protein which is toxic to *E. coli *and rapidly degraded at 37°C was placed under the control of the *cspA *promoter. Temperature downshift to 15 or 23°C abolished degradation of the fusion protein and the toxic phenotype associated with expression at 37°C was suppressed. It was suggested that this system is a valuable tool for the production of proteins containing membrane-spanning domains or otherwise unstable gene products in *E. coli*.

A principle that allows for protein expression and folding at 4°C was presented recently [17]. This principle is based on co-expression of the target protein with chaperones from a psychrophilic bacterium. The two chaperones (Cpn60 and Cpn10 from *Oleispira antarctica *RB8^T^) allow *E. coli *to grow at high rates at 4°C [12]. An esterase from *O. antarctica *RB8^T ^was co-expressed with Cpn60 and Cpn10 in *E. coli *at 4°C. This procedure increased the specific activity of the purified esterase 180 fold as compared to enzyme prepared from cultivations at 37°C. It was concluded that the low temperature was beneficial to folding and the system was suggested as a tool for expression and correct folding of recombinant proteins in the cytoplasm of *E. coli*.

### *E. coli *strains used to improve soluble expression

Numerous specialized host strains have been developed to overcome the metabolic burden related to high level protein expression.

Two *E. coli *mutant strains have contributed significantly to the soluble expression of difficult recombinant proteins. C41(DE3) and C43(DE3) are mutants that allow over-expression of some globular and membrane proteins unable to be expressed at high-levels in the parent strain BL21(DE3) [18]. Expression of the F_1_F_o _ATP synthase subunit b membrane protein in these strains, in particular C43(DE3), is accompanied by the proliferation of intracellular membranes and inclusion bodies are absent [19]. These strains are now commercialized by Avidis  and a high number of reports on their use in expression of difficult proteins have been published [20-23]. A recent work reports that the stability of plasmids encoding toxic proteins is increased in C41(DE3) and especially in C43(DE3) [24].

Cysteines in the *E. coli *cytoplasm are actively kept reduced by pathways involving thioredoxin reductase and glutaredoxin. The disulfide bond dependent folding of heterologous proteins is improved in the Origami strains from Novagen. Disruption of the *trxB *and *gor *genes encoding the two reductases, allow the formation of disulfide bonds in the *E. coli *cytoplasm. The *trxB *(Novagen AD494) and *trxB/gor *(Novagen Origami) negative strains of *E. coli *have been selected in several expression situations [25-27]. Folding and disulfide bond formation in the target protein, is enhanced by fusion to thioredoxin in strains lacking thioredoxin reductase (*trxB*) [28]. Overexpression of the periplasmic foldase DsbC in the cytoplasm stimulates disulfide bond formation further [27].

### Modification of cultivation strategies to obtain soluble protein

The simplest way to produce a recombinant protein is by batch cultivation. Here all nutrients required for growth are supplied from the beginning and there is a limited control of the growth during the process. This limitation often leads to changes in the growth medium such as changes in pH and concentration of dissolved oxygen as well as substrate depletion. Furthermore inhibitory products of various metabolic pathways accumulate. Cell densities and production levels are only moderate in batch cultivations.

In fed batch cultivations, the concentration of energy sources can be adjusted according to the rate of consumption. Several other factors can also be regulated in order to obtain the maximal production level in terms of target protein per biomass. The formation of inclusion bodies can be followed in fed batch cultivations by monitoring changes in intrinsic light scattering by flow cytometry [29]. This allows for real time optimization of growth conditions as soon as inclusion bodies are detected even at low levels and inclusion body formation can potentially be avoided [30].

Folding of some proteins require the existence of a specific cofactor. Addition of such cofactors or binding partners to the cultivation media may increase the yield of soluble protein dramatically. This was demonstrated for a recombinant mutant of hemoglobin for which the accumulation of soluble product was improved when heme was in excess [31]. Similarly, a 50% increase in solubility was observed for gloshedobin when *E. coli *recombinants were cultivated in the presence of 0.1 mM Mg^2+ ^[32]. An important factor in soluble expression of recombinant proteins is media composition and optimization. Although this is attained mostly by trial and error, it nevertheless may be beneficial.

### Molecular chaperones drive folding of recombinant proteins

A possible strategy for the prevention of inclusion body formation is the co-overexpression of molecular chaperones. This strategy is attractive but there is no guarantee that chaperones improve recombinant protein solubility. *E. coli *encode chaperones, some of which drive folding attempts, whereas others prevent protein aggregation [4,11,33]. As soon as newly synthesized proteins leave the exit tunnel of the *E. coli *ribosome they associate with the trigger factor chaperone [34]. Exposed hydrophobic patches on newly synthesized proteins are protected by association with trigger factor from unintended inter- or intramolecular interactions thus preventing premature folding. Proteins can start or continue their folding into the native state after release from trigger factor. Proteins trapped in non-native and aggregation prone conformations, are substrates for DnaK and GroEL. DnaK (Hsp70 chaperone family) prevents the formation of inclusion bodies by reducing aggregation and promoting proteolysis of misfolded proteins [11]. A bi-chaperone system involving DnaK and ClpB (Hsp100 chaperone family) mediates the solubilization or disaggregation of proteins [35]. GroEL (Hsp60 chaperone family) operates the protein transit between soluble and insoluble protein fractions and participates positively in disaggregation and inclusion body formation. Small heat shock proteins lbpA and lbpB protect heat denatured proteins from irreversible aggregation and have been found associated with inclusion bodies [36,37].

Simultaneous over-expression of chaperone encoding genes and recombinant target proteins proved effective in several instances. Co-overexpression of trigger factor in recombinants prevented the aggregation of mouse endostatin, human oxygen-regulated protein ORP150, human lysozyme and guinea pig liver transglutaminase [38,39]. Soluble expression was further stimulated by the co-overexpression of the GroEL-GroES and DnaK-DnaJ-GrpE chaperone systems along with trigger factor [39]. The chaperone systems are cooperative and the most favorable strategies involve co-expression of combinations of chaperones belonging to the GroEL, DnaK, ClpB and ribosome associated trigger factor families of chaperones [40-42].

### Interaction partners and protein folding

Protein insolubility in the *E. coli *cytoplasm is partially related to the distribution of hydrophobic residues on the surface of the protein. The soluble expression of subunits of hetero multimeric proteins therefore sometimes suffers from inclusion body formation in the absence of an appropriate binding partner.

Soluble expression in *E. coli *of the bacteriophage T4 gene 23 product (major capsid protein) required the co-expression of gene product 31 (phage co-chaperonin gp31) [43]. Expression of the correct interaction partner enabled gp23 to fold correctly and form long regular structures in the cytoplasm of *E. coli*.

Another study reports the purification of a heterodimeric complex by expression of each subunit (pheromaxein A and C) as a fusion to thioredoxin [44]. Each subunit remained soluble in solution, when thioredoxin was proteolytically removed, only in the presence of the other.

Conclusively, interaction partners potentially favour *in vivo *solubility of target proteins. New systems for co-expression of multiple proteins involved in complex structures enable such strategies [1].

## Strategies involving engineered target protein

Target proteins are not always expressed in a soluble form by the strategies described above. The last part of this review discusses how misfolded proteins can be engineered or pushed to evolve and selected to gain soluble expression.

### Fusion protein technology

The use of affinity tags in recombinant protein purification has a long tradition. Not only have they been exploited for the development of generic purification strategies. Affinity tags have been observed to improve protein yield, to prevent proteolysis and to increase solubility *in vivo *[1,45].

Among the most potent solubility enhancing proteins characterized to date are the *E. coli *maltose binding protein (MBP) and the *E. coli *N-utilizing substance A (NusA). MBP (40 kDa) and NusA (54.8 kDa) act as solubility enhancing partners and are especially suited for the expression of proteins prone to form inclusion bodies. Although many proteins are highly soluble, they are not all effective as solubility enhancers. *E. coli *MBP proved to be a much more effective solubility partner than the highly soluble GST and thioredoxin proteins in a comparison of solubility enhancing properties [46]. Solubility enhancement is a common trait of maltodextrin-binding proteins (MBPs) from a number of organisms and some of them are even more effective than *E. coli *MBP [47]. A precise mechanism for the solubility enhancement of MBP has not been found. However, MBP might act as a chaperone by interactions through a solvent exposed "hot spot" on its surface which stabilizes the otherwise insoluble passenger protein [48,49]. The ability of MBP to promote the solubility of fusion partners can be improved by addition of supplemental tags. Different configurations for MBP fusion proteins have been suggested for high-throughput protein expression and purification [50].

Wilkinson and Harrison proposed a model for the theoretical calculation of solubility percentages of recombinant proteins expressed in the *E. coli *cytoplasm [51]. A webserver for the calculation of this index is found at . The Wilkinson-Harrison model along with experimental data identified NusA as a highly favorable solubility partner [52]. The major advantage of NusA, in addition to the good solubility characteristics, is its high expressivity. Both MBP and NusA have been used for the solubilization of highly insoluble ScFv antibodies in the cytoplasm of *E. coli *[48,53]. Numerous examples of MBP and NusA as functional solubility enhancers are found in the literature [54-57].

Natural molecular chaperones that have been used as solubility enhancers include prolyl *cis trans *isomerases (PPIases) [58], thioredoxin [59] and dsbA [60].

Fusion partners such as MBP and NusA are relatively large proteins. We recently suggested the use of a highly soluble N-terminal fragment of translation initiation factor IF2 (17.4 kDa) as a solubility partner [61]. The use of a small partner reduces the amount of energy required to obtain a certain number of molecules, diminishes steric hindrance and simplify downstream applications such as NMR. Another relatively small protein, barnase was suggested to exert chaperone like functions both *in vivo *and *in vitro *when fused to the C-terminus of the light chain variable domain of an IgG [62].

In a recent study it was shown that a 17 residue C-terminal extension of Pfg27 resulted in several fold enhancement of soluble expression [63]. Several studies have shown that the nature of terminal residues in proteins can play a role in recognition and subsequent action by proteases [64,65]. The terminal extension of proteins might therefore indirectly protect them from the denaturaturation/misfolding associated to partial proteolytic degradation. It has also been suggested that large net charges of peptide extensions increases electrostatic repulsion between nascent polypeptides and therefore enhances their correct folding [66].

Screening strategies have been employed to select for favorable fusion partners in a high throughput manner. In such a system more than 80% of the proteins tested showed high levels of expression of soluble products with at least one of eight fusion partners including NusA, intein, thioredoxin, His-tag, MBP, calmodulin binding protein and glutathione-S-transferase [67]. These results were supported by another similar study [68].

### Screening for and selection of soluble variants

Structural and functional genomics and proteomics are important elements in the evaluation of gene function. The expression and purification of properly folded proteins in a high throughput manner are key elements in these studies. A number of different approaches to the high throughput screening of soluble expression products have been described recently.

The intrinsic folding yield, stability and solubility of target proteins can be improved by engineering the target protein. When structural information is available, the solubility of the expressed protein has been improved by rational site directed mutagenesis [69]. A more general approach is to find more soluble variants by directed evolution. Libraries generated in this context include random point mutants, deletions and fragments [70]. The generated mutants are screened for solubility either by the function of the protein of interest or by more general screens. A screen based on biological activity implies that a new assay has to be developed for every new protein studied. Moreover, in many cases the protein or protein domain studied does not display any known activity at all. The general screens include fusion reporter methods, stress reporter methods and direct methods and are therefore usually preferred for high-throughput approaches.

Fluorescence of *E. coli *cells expressing target genes fused to the GFP-gene is related to the solubility of the target gene expressed alone [71]. Hence, protein folding in *E. coli *can be improved by directed evolution approaches for a certain target protein by screening for fluorescing mutants. This approach evolved three insoluble proteins including *Pyrobaculum aerophilum *methyl transferase, tartrate dehydratase β-subunit and nucleoside diphosphate kinase to be 50%, 95% and 90% soluble respectively [72]. The GFP reporter system was further used to screen for solubilizing interaction partners to insoluble targets. Fusion of integration host factor β upstream to GFP resulted in aggregation, whereas co-expression of the binding partner (integration host factor α) increased fluorescence dramatically [73].

A similar approach is the use of selective pressure. By fusing target proteins with chloramphenicol acetyl transferase (CAT) more soluble fusion protein mutants were selected on media containing progressively higher levels of chloramphenicol [74]. Furthermore, selective pressure (fusion to kanamycin phosphotransferase) was used in a system aiming at the obtainment of soluble proteins encoded by cDNA fragments in a high throughput approach [75].

Another fusion reporter method use the β-galactosidase α peptide as fusion partner in a screen for lacZα complementation in a system where inactive lacZΩ is supplied *in trans*. Active β-galactosidase can be detected when the α peptide becomes soluble and restore enzyme activity by binding to lacZΩ [76].

An innate host cell response is induced when recombinantly expressed proteins are misfolded. This response can be monitored by the transcription from *E. coli *promoters that are up-regulated when misfolded proteins are expressed. It was found that the promoter for the small heat shock protein ibpA could be fused to lacZ and used as a reporter for misfolded protein [77]. This reporter could discriminate soluble, partially soluble and insoluble recombinant proteins. Genetic screens and directed evolution is further reviewed elsewhere [78].

Soluble fusion proteins are not necessarily biologically active and properly folded. Several reports have demonstrated that soluble preparations of fusion proteins have low biological activity as compared to the non-fused protein [79]. It was shown that a fusion of HPV oncoprotein E6 to MBP formed soluble multimeric aggregates composed of folded MBP and misfolded E6. These "soluble inclusion bodies" could be avoided by optimization of the expression conditions by screening for monodispersity [79].

### Alternative strategies

A few strategies that are radically different from the conventional fusion partner and selection approaches have been developed for the potential rescuing of recombinant proteins from misfolding in the *E. coli *cytoplasm.

A system based on artificial oil bodies was developed and illustrated by a fusion protein composed of oleosin and GFP [80]. The expressed fusion protein was found in the insoluble cellular fraction but could be reconstituted as oil-bodies by addition of triacylglycerol and phospholipids to the purified inclusion bodies. GFP could subsequently be separated from the oil bodies using an engineered factor Xa cleavage site and centrifugation.

An *in vivo *rescuing system based on the *E. coli *ribosome was recently presented [81]. Target proteins are rescued from *in vivo *aggregation by fusing them to ribosomal protein L23. The fusion protein is expressed in a strain of *E. coli *deficient in the essential L23 ribosomal protein. This allows for the covalent coupling of target proteins to the highly soluble ribosomal particles. Ribosomes with coupled target protein can subsequently be isolated by centrifugation methods and the target protein released in a highly enriched form by site specific protease cleavage.

## Conclusions

We have reviewed the most recent improvements in obtaining soluble and functional protein preparations from *E. coli *recombinants. A subset of the methods focus on relieving the cellular stress that is a response to the extreme metabolic situation experienced by the host cell during the process of hyperexpression of a single or a few proteins. A second subset of methods focus on improving the solubility and structural stability of the expressed protein, by the combination of the target protein with specific peptide tags. A common trait in modern expression strategies is the skillful combination of the utensils in the genetic toolbox, but also a constant reconsideration of the accepted paradigms in trade of protein expression.

## References

[B1] Sørensen HP, Mortensen KK (2005). Advanced genetic strategies for recombinant expression in Escherichia coli. J Biotechnol.

[B2] Villaverde A, Carrio MM (2003). Protein aggregation in recombinant bacteria: biological role of inclusion bodies. Biotechnol Lett.

[B3] Anfinsen CB (1973). Principles that govern the folding of protein chains. Science.

[B4] Hartl FU, Hayer-Hartl M (2002). Molecular chaperones in the cytosol: From nascent chain to folded protein. Science.

[B5] Middelberg A (2002). Preparative protein refolding. Trends Biotechnol.

[B6] Bentley WE, Kompala DS (1990). Optimal induction of protein synthesis in recombinant bacterial cultures. Ann N Y Acad Sci.

[B7] Schein CH (1989). Production of soluble recombinant proteins in bacteria. BioTechnology.

[B8] Vasina JA, Baneyx F (1997). Expression of aggregation prone recombinant proteins at low temperatures: a comparative study of the Escherichia coli cspA and tac promoter systems. Protein Expr Purif.

[B9] Kiefhaber T, Rudolph R, Kohler HH, Buchner J (1991). Protein aggregation in vitro and in vivo: a quantitative model of the kinetic competition between folding and aggregation. Biotechnology (N Y).

[B10] Chesshyre JA, Hipkiss AR (1989). Low temperatures stabilize interferon a-2 against proteolysis in Methylophilus methylotrophus and Escherichia coli. Appl Microbiol Biotechnol.

[B11] Mogk A, Mayer MP, Deuerling E (2002). Mechanisms of protein folding: molecular chaperones and their application in biotechnology. Chembiochem.

[B12] Ferrer M, Chernikova TN, Yakimov MM, Golyshin PN, Timmis KN (2003). Chaperonins govern growth of Escherichia coli at low temperature. Nat Biotechnol.

[B13] Shaw MK, Ingraham JL (1967). Synthesis of macromolecules by Escherichia coli near the minimal temperature for growth. J Bacteriology.

[B14] Vasina JA, Baneyx F (1996). Recombinant protein expression at low temperatures under the transcriptional control of the major Escherichia coli cold shock promoter cspA. Appl Environ Microbiol.

[B15] Weickert MJ, Doherty DH, Best EA, Olins PO (1996). Optimization of heterologous protein production in Escherichia coli. Curr Opin Biotechnol.

[B16] Mujacic M, Cooper KW, Baneyx F (1999). Cold-inducible cloning vectors for low-temperature protein expression in Escherichia coli: application to the production of a toxic and proteolytically sensitive fusion protein. Gene.

[B17] Ferrer M, Chernikova TN, Timmis KN, Golyshin PN (2004). Expression of a temperature sensitive esterase in a novel chaperone based Escherichia coli strain. Appl Environ Microbiol.

[B18] Miroux B, Walker JE (1996). Over-production of proteins in Escherichia coli: mutant hosts that allow synthesis of some membrane proteins and globular proteins at high levels. J Mol Biol.

[B19] Arechaga I, Miroux B, Karrasch S, Huijbregts R, de Kruijff B, Runswick MJ, Walker JE (2000). Characterisation of new intracellular membranes in Escherichia coli accompanying large scale over-production of the b subunit of F(1)F(o) ATP synthase. FEBS Lett.

[B20] Steinfels E, Orelle C, Dalmas O, Penin F, Miroux B, Di Pietro A, Jault JM (2002). Highly efficient over-production in E. coli of YvcC, a multidrug-like ATP-binding cassette transporter from Bacillus subtilis. Biochim Biophys Acta.

[B21] Smith VR, Walker JE (2003). Purification and folding of recombinant bovine oxoglutarate/malate carrier by immobilized metal-ion affinity chromatography. Protein Expr Purif.

[B22] Arechaga I, Miroux B, Runswick MJ, Walker JE (2003). Over-expression of Escherichia coli F1F(o)-ATPase subunit a is inhibited by instability of the uncB gene transcript. FEBS Lett.

[B23] Sørensen HP, Sperling-Petersen HU, Mortensen KK (2003). Production of recombinant thermostable proteins expressed in Escherichia coli: completion of protein synthesis is the bottleneck. J Chromatogr B.

[B24] Dumon-Seignovert L, Cariot G, Vuillard L (2004). The toxicity of recombinant proteins in Escherichia coli: a comparison of overexpression in BL21(DE3), C41(DE3), and C43(DE3). Protein Expr Purif.

[B25] Lehmann K, Hoffmann S, Neudecker P, Suhr M, Becker WM, Rosch P (2003). High-yield expression in Escherichia coli, purification, and characterization of properly folded major peanut allergen Ara h 2. Protein Expr Purif.

[B26] Premkumar L, Bageshwar UK, Gokhman I, Zamir A, Sussman JL (2003). An unusual halotolerant alpha-type carbonic anhydrase from the alga Dunaliella salina functionally expressed in Escherichia coli. Protein Expr Purif.

[B27] Bessette PH, Aslund F, Beckwith J, Georgiou G (1999). Efficient folding of proteins with multiple disulfide bonds in the Escherichia coli cytoplasm. Proc Natl Acad Sci U S A.

[B28] Stewart EJ, Aslund F, Beckwith J (1998). Disulfide bond formation in the Escherichia coli cytoplasm: an in vivo role reversal for the thioredoxins. EMBO J.

[B29] Fouchet P, Manin C, Richard H, Frelat G, Barbotin JN (1994). Flow cytometry studies of recombinant Escherichia coli in batch and continous cultures: DNA and RNA contents; light scatter parameters. Appl Microbiol Biotechnol.

[B30] Lewis G, Taylor IW, Nienow AW, Hewitt CJ (2004). The application of multi-parameter flow cytometry to the study of recombinant Escherichia coli batch fermentation processes. J Ind Microbiol Biotechnol.

[B31] Weickert MJ, Pagratis M, Glascock CB, Blackmore R (1999). A mutation that improves soluble recombinant hemoglobin accumulation in Escherichia coli in heme excess. Appl Environ Microbiol.

[B32] Yang Q, Xu J, Li M, Lei X, An L (2003). High-level expression of a soluble snake venom enzyme, gloshedobin, in E. coli in the presence of metal ions. Biotechnol Lett.

[B33] Schwarz E, Lilie H, Rudolph R (1996). The effect of molecular chaperones on in vivo and in vitro folding processes. Biol Chem.

[B34] Deuerling E, Patzelt H, Vorderwulbecke S, Rauch T, Kramer G, Schaffitzel E, Mogk A, Schulze-Specking A, Langen H, Bukau B (2003). Trigger Factor and DnaK possess overlapping substrate pools and binding specificities. Mol Microbiol.

[B35] Schlieker C, Bukau B, Mogk A (2002). Prevention and reversion of protein aggregation by molecular chaperones in the E. coli cytosol: implications for their applicability in biotechnology. J Biotechnol.

[B36] Kuczynska-Wisnik D, Kedzierska S, Matuszewska E, Lund P, Taylor A, Lipinska B, Laskowska E (2002). The Escherichia coli small heat-shock proteins IbpA and IbpB prevent the aggregation of endogenous proteins denatured in vivo during extreme heat shock. Microbiology.

[B37] Kitagawa M, Miyakawa M, Matsumura Y, Tsuchido T (2002). Escherichia coli small heat shock proteins, IbpA and IbpB, protect enzymes from inactivation by heat and oxidants. Eur J Biochem.

[B38] Ikura K, Kokubu T, Natsuka S, Ichikawa A, Adachi M, Nishihara K, Yanagi H, Utsumi S (2002). Co-overexpression of folding modulators improves the solubility of the recombinant guinea pig liver transglutaminase expressed in Escherichia coli. Prep Biochem Biotechnol.

[B39] Nishihara K, Kanemori M, Yanagi H, Yura T (2000). Overexpression of trigger factor prevents aggregation of recombinant proteins in Escherichia coli. Appl Environ Microbiol.

[B40] Amrein KE, Takacs B, Stieger M, Molnos J, Flint NA, Burn P (1995). Purification and characterization of recombinant human p50csk protein-tyrosine kinase from an Escherichia coli expression system overproducing the bacterial chaperones GroES and GroEL. Proc Natl Acad Sci U S A.

[B41] Nishihara K, Kanemori M, Kitagawa M, Yanagi H, Yura T (1998). Chaperone coexpression plasmids: differential and synergistic roles of DnaK-DnaJ-GrpE and GroEL-GroES in assisting folding of an allergen of Japanese cedar pollen, Cryj2, in Escherichia coli. Appl Environ Microbiol.

[B42] Held D, Yaeger K, Novy R (2003). New coexpression vectors for expanded compatibilities in E. coli. InNovations.

[B43] Kurochkina LP, Mesyanzhinov VV (1999). Co-expression of Gene 31 and 23 products of Bacteriophage T4. Biochemistry (Mosc).

[B44] Austin C (2003). Novel approach to obtain biologically active recombinant heterodimeric proteins in Escherichia coli. J Chromatogr B Analyt Technol Biomed Life Sci.

[B45] Makrides SC (1996). Strategies for achieving high-level expression of genes in Escherichia coli.. Microbiol Rev.

[B46] Kapust RB, Waugh DS (1999). Escherichia coli maltose-binding protein is uncommonly effective at promoting the solubility of polypeptides to which it is fused. Protein Sci.

[B47] Fox JD, Routzahn KM, Bucher MH, Waugh DS (2003). Maltodextrin-binding proteins from diverse bacteria and archaea are potent solubility enhancers. FEBS Lett.

[B48] Bach H, Mazor Y, Shaky S, Shoham-Lev A, Berdichevsky Y, Gutnick DL, Benhar I (2001). Escherichia coli maltose-binding protein as a molecular chaperone for recombinant intracellular cytoplasmic single-chain antibodies. J Mol Biol.

[B49] Fox JD, Kapust RB, Waugh DS (2001). Single amino acid substitutions on the surface of Escherichia coli maltose-binding protein can have a profound impact on the solubility of fusion proteins. Protein Sci.

[B50] Routzahn KM, Waugh DS (2002). Differential effects of supplementary affinity tags on the solubility of MBP fusion proteins. J Struct Funct Genomics.

[B51] Wilkinson DL, Harrison RG (1991). Predicting the solubility of recombinant proteins in Escherichia coli. Biotechnology (N Y).

[B52] Davis GD, Elisee C, Newham DM, Harrison RG (1999). New fusion protein systems designed to give soluble expression in Escherichia coli. Biotechnol Bioeng.

[B53] Zheng L, Baumann U, Reymond JL (2003). Production of a functional catalytic antibody ScFv-NusA fusion protein in bacterial cytoplasm. J Biochem (Tokyo).

[B54] Elizeev R, Alexandrov A, Gunter T (2004). High-yield expression and purification of p18 form of Bax as an MBP fusion protein. Protein Expr Purif.

[B55] Smyth DR, Mrozkiewicz MK, McGrath WJ, Listwan P, Kobe B (2003). Crystal structures of fusion proteins with large affinity tags. Protein Sci.

[B56] Ermolova NV, Cushman MA, Taybi T, Condon SA, Cushman JC, Chollet R (2003). Expression, purification, and initial characterization of a recombinant form of plant PEP-carboxylase kinase from CAM induced Mesembryanthemum crystallinum with enhanced solubility in Escherichia coli. Protein Expr Purif.

[B57] Goh LL, Loke P, Singh M, Sim TS (2003). Soluble expression of functionally active Plasmodium falciparum falcipain-2 fused to maltose-binding protein in Escherichia coli. Protein Expr Purif.

[B58] Ideno A, Furutani M, Iwabuchi T, Iida T, Iba Y, Kurosawa Y, Sakuraba H, Ohshima T, Kawarabayshi Y, Maruyama T (2004). Expression of foreign proteins in Escherichia coli by fusing with an archaeal FK506 binding protein. Appl Microbiol Biotechnol.

[B59] Jacquet A, Daminet V, Haumont M, Garcia L, Chaudoir S, Bollen A, Biemans R (1999). Expression of a recombinant Toxoplasma gondii ROP2 fragment as a fusion protein in bacteria circumvents insolubility and proteolytic degradation. Protein Expr Purif.

[B60] Zhang Y, Olsen DR, Nguyen KB, Olson PS, Rodes ET, Mascarenhas D (1998). Expression of eukaryotic proteins in soluble form in Escherichia coli. Protein Expr Purif.

[B61] Sørensen HP, Sperling-Petersen HU, Mortensen KK (2003). A favorable solubility partner for the recombinant expression of streptavidin. Protein Expr Purif.

[B62] Matsev SP, Tsybovsky YI, Stremovskiy OA, Odintsov SG, Balandin GT, Arosio P, Kravchuk ZI, Deyev SM (2004). Fusion of the antiferritin antibody VL domain to barnase results in enhanced solubility and pH stability. Protein Eng Des Sel.

[B63] Sati SP, Singh SK, Kumar N, Sharma A (2002). Extra terminal residues have profound effect on the folding and solubility of a Plasmodium falciparum sexual stage-specific protein over-expressed in Escherichia coli. Eur J Biochem.

[B64] Silber KR, Keiler KC, Sauer RT (1992). Tsp: a tail-specific protease that selectively degrades proteins with nonpolar C-termini. Proc Natl Acad Sci U S A.

[B65] Bowie JU, Sauer RT (1989). Identification of C-terminal extensions that protect poteins from intracellular proteolysis. J Biol Chem.

[B66] Zhang Y, Howitt J, McCorkle S, Lawrence P, Springer K, Freimuth P (2004). Protein aggregation during overexpression limited by peptide extensions with large net negative charge. Protein Expr Purif.

[B67] Shi PY, Kung WM, Chen JC, Yeh CH, Wang AHJ, Wang TF (2002). High-throughput screening of soluble recombinant proteins. Protein Sci.

[B68] Hammarström M, Hellgren N, van den Berg S, Berglund H, Härd T (2002). Rapid screening for improved solubility of small human proteins produced as fusion proteins in Escherichia coli. Protein Sci.

[B69] Dale GE, Broger C, Langen H, D´arcy A, Stuber D (1994). Improving protein solubility through rationally designed amino acid replacements. Protein Eng.

[B70] Farinas ET, Butler T, Arnold F (2001). Directed enzyme evolution. Curr Opin Biotechnol.

[B71] Waldo GS, Standish BM, Berendzen J, Terwilliger TC (1999). Rapid protein-folding assay using green fluorescent protein. Nat Biotechnol.

[B72] Pedelacq JD, Piltch E, Liong EC, Berendzen J, Kim CY, Rho BS, Park MS, Terwilliger TC, Waldo GS (2002). Engineering soluble proteins for structural genomics. Nat Biotechnol.

[B73] Wang H, Chong S (2003). Visualization of coupled protein folding and binding in bacteria and purification of the heterodimeric complex. Proc Natl Acad Sci U S A.

[B74] Maxwell KL, Mittermaier AK, Forman-Kay JD, Davidson AR (1999). A simple in vivo assay for increased protein solubility. Protein Sci.

[B75] Nakayama M, Ohara O (2003). A system using convertible vectors for screening soluble recombinant proteins produced in Escherichia coli from randomly fragmented cDNAs. Biochem Biophys Res Commun.

[B76] Wigley WC, Stidham RD, Smith NM, Hunt JF, Thomas PJ (2001). Protein solubility and folding monitored in vivo by structural complementation of a genetic marker protein. Nat Biotechnol.

[B77] Lesley SA, Graziano J, Cho CY, Knuth MW, Klock HE (2002). Gene expression response to misfolded protein as a screen for soluble recombinant protein. Protein Eng.

[B78] Waldo GS (2003). Genetic screens and directed evolution for protein solubility. Curr Opin Chem Biol.

[B79] Nomine Y, Ristriani T, Laurent C, Lefevre J, Weiss E, Trave G (2001). A strategy for optimizing the mondispersity of fusion proteins: application to purification of recombinant HPV E6 oncoprotein. Protein Eng.

[B80] Peng C, Chen JCF, Shyu DJH, Chen M, Tzen JTC (2004). A system for purification of recombinant proteins in Escherichia coli via artificial oil bodies constituted with their oleosin fused polypeptides. J Biotechnol.

[B81] Sørensen HP, Kristensen JE, Sperling-Petersen HU, Mortensen KK (2004). Soluble expression of aggregating proteins by covalent coupling to the ribosome. Biochem Biophys Res Commun.

